# Outcome of extensive varus and valgus stem alignment in short-stem THA: clinical and radiological analysis using EBRA-FCA

**DOI:** 10.1007/s00402-017-2640-z

**Published:** 2017-02-02

**Authors:** Karl Philipp Kutzner, Tobias Freitag, Stefanie Donner, Mark Predrag Kovacevic, Ralf Bieger

**Affiliations:** 1 Department of Orthopaedic Surgery and Traumatology, St. Josefs Hospital Wiesbaden, Beethovenstr. 20, 65189 Wiesbaden, Germany; 2 Department of Orthopaedic Surgery, University of Ulm, Oberer Eselsberg 45, 89081 Ulm, Germany; 3 Department of Traumatology, Hand- and Orthopaedic Surgery, HELIOS Dr. Horst Schmidt Clinic Wiesbaden, Ludwig-Erhard-Str. 100, 65199 Wiesbaden, Germany

**Keywords:** Total hip arthroplasty, Short stem, Stem alignment, Optimys, EBRA, Cortical hypertrophy, Stress-shielding, Varus, Valgus

## Abstract

**Introduction:**

The principle of implanting a calcar-guided short stem consists of an individual alignment alongside the medial calcar providing the ability of reconstructing varus and valgus anatomy in a great variety. However, still, there are broad concerns about the safety of extensive varus and valgus positioning in regard to stability, bony alterations, and periprosthetic fractures.

**Materials and methods:**

216 total hip arthroplasties using a calcar-guided short stem (optimys, Mathys Ltd.) in 162 patients were included. Depending on postoperative CCD angle, hips were divided into five groups (A–E). Varus- and valgus tilt and axial subsidence were assessed by “Einzel-Bild-Roentgen-Analyse”(EBRA-FCA, femoral component analysis) over a 2-year follow-up. The incidence of stress-shielding and cortical hypertrophy as well as clinical outcome [Harris Hip Score (HHS)] were reported.

**Results:**

Postoperative CCD angles ranged from 117.9° to 145.6° and mean postoperative CCD angles in group A–E were 123.3°, 128.0°, 132.4°, 137.5°, and 142.5°, respectively. After 2 years, the mean varus/valgus tilt was −0.16°, 0.37°, 0.48°, 0.01°, and 0.86°, respectively (*p* = 0.502). Axial subsidence after 2 years was 1.20, 1.02, 1.44, 1.50, and 2.62 mm, respectively (*p* = 0.043). No periprosthetic fractures occurred and none of the stems had to be revised. Rates of stress-shielding and cortical hypertrophy as well as HHS showed no significant difference between the groups.

**Conclusions:**

Valgus alignment results in increased subsidence but does not affect the clinical outcome. There is no difference in stress shielding and cortical hypertrophy between the groups. The authors recommend long term monitoring of valgus aligned stems.

## Introduction

In modern primary total hip arthroplasty (THA), short stems are increasingly regarded as implants of first choice [1], especially in young and active patients [2, 3]. All short-stem designs aim at the preservation of proximal bone by reducing stress-shielding due to periprosthetic bone remodelling [4, 5]. However, shortening the femoral stem and reducing the diaphyseal fixation might lead to a possible reduction of stability and lead to changes in the migration pattern [6]. Both, stress-shielding and implant migration, are considered to possibly cause aseptic loosening in cementless THA [5, 7].

In the last decade, many different short-stem designs have been introduced [8, 9], resulting in a highly heterogenic group. Clinical mid-term results of many designs are encouraging [2, 10–12]. However, also certain designs have already been withdrawn from the market [13].

**Fig. 1:**
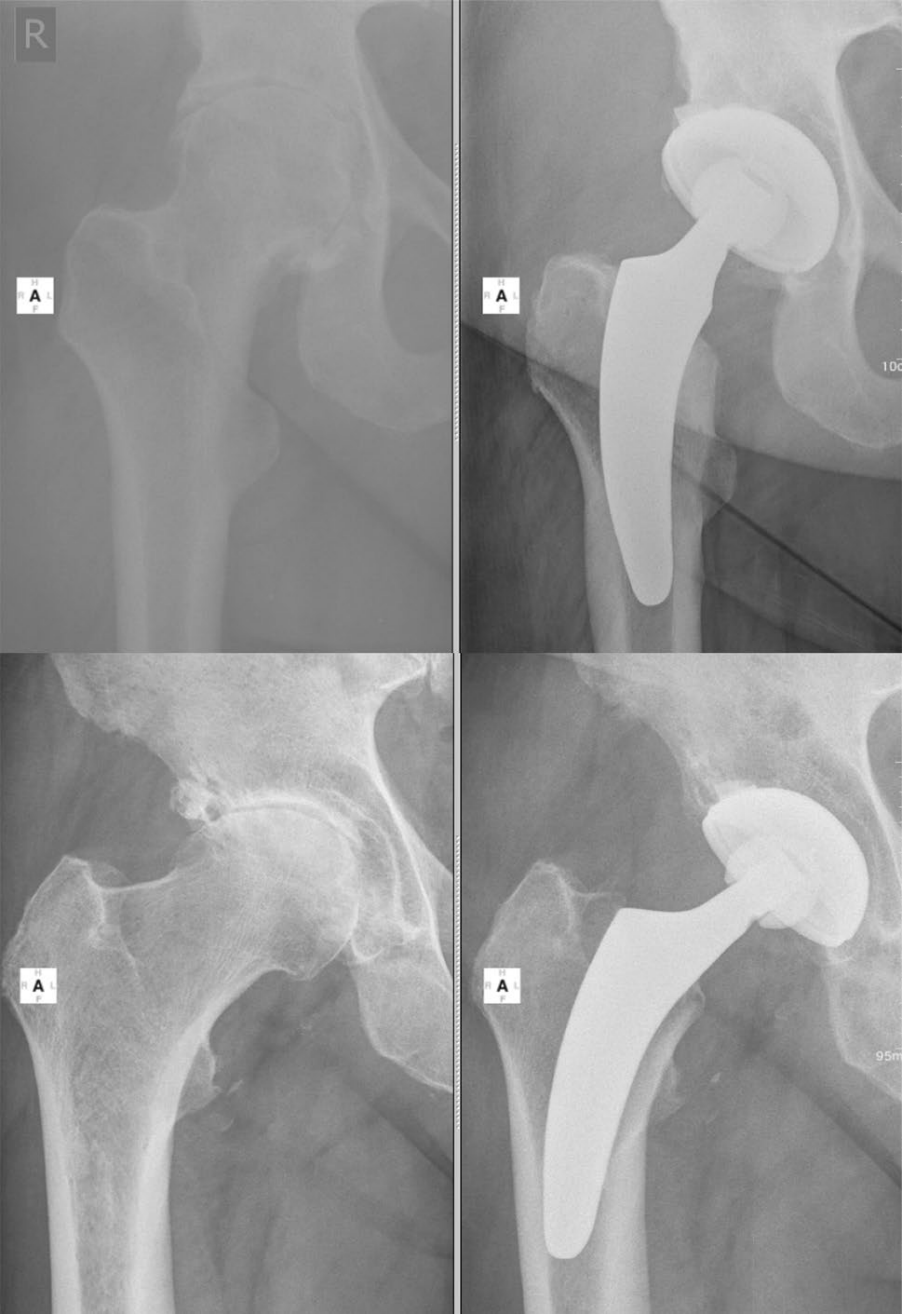
Possible extensive varus- and valgus stem alignments result in a broad range of CCD angles to be reconstructed with the investigated short stem. *Upper row* extensive valgus alignment (*left* preop, *right* 2-year follow-up); *lower row* extensive varus alignment (*left* preop, *right* 2-year follow-up)

So-called calcar-guided short stems have been developed to optimally adapt to the anatomy of the proximal femur and to allow a restoration of hip biomechanics [14, 15]. They align themselves alongside the medial cortical bone, sparing the greater trochanter completely [16]. Implantation is done in a “round-the-corner” technique, using individualized levels of osteotomy to align the stem in varus- or valgus position, according to the patient’s anatomy [14, 15]. This results in a broad range of CCD angles to be reconstructed with these types of stems [15, 17] with possible extensive varus- and valgus alignments (Fig. 1). Calcar-guided short stems achieve stabilization by metaphyseal anchoring, based on the fit-and-fill principle. However, three-point anchoring is possible in some cases.

To date, little is known about the outcome of extensive varus- or valgus positioning in calcar-guided short-stem THA. There still are broad concerns particularly regarding implant stability. It might have an impact on the rate of axial subsidence as well as on stem tilting. In addition, the influence of different stem alignment on bony alterations like stress-shielding and cortical hypertrophy, as a result of abnormal stress distribution in the loaded proximal femur, to date, is not fully understood. Consequently, it remains unclear that how different stem alignments affect stability and load transfer.

The objective of the present analysis was to address the following research questions:

- Does extensive varus- and valgus alignment in calcar-guided short-stem THA impact on postoperative varus- and valgus tilt and subsidence?
- Does different stem alignment affect the incidence of stress-shielding and cortical hypertrophy?
- Is there a difference in postoperative functional outcome?

## Materials and methods

In the present retrospective investigation, 216 consecutive hips in 162 patients were included after institutional review board approval (University of Ulm, Germany, 323/13) from an ongoing prospective observational study. Preliminary results of the same study group have already been published involving the same collective of patients in a different context [18, 19]. Prior to inclusion, written consent to participate has been obtained from all patients. Between 2010 and 2012, 74 women and 88 men were operated using the investigated calcar-guided short-stem. In 54 patients, the treatment was one stage bilaterally; 108 patients were operated unilaterally. The indications for implantation were: 91.7% (*n* = 198) primary osteoarthrosis, 5.1% (*n* = 11) femoral head necrosis, 2.3% (*n* = 5) congenital dysplasia, and 0.9% (*n* = 2) secondary osteoarthrosis. Mean patient age was 63.0 years (standard deviation (SD) 10.0).

In all patients, the meta-diaphyseal anchoring calcar-guided short stem optimys (Mathys Ltd., Bettlach, Switzerland) was implanted as described in Kutzner et al. [18, 19].

The investigated stem was combined with cementless press-fit cups (*n* = 177 Fitmore, Zimmer; *n* = 39 RM Pressfit vitamys, Mathys Ltd Bettlach) with a ceramic-polyethylene bearing couple. All surgeries were performed in supine position using a modified, minimally invasive anterolateral approach [20]. Full weight-bearing using two crutches was allowed in all cases immediately after surgery.

All patients underwent pre- and postoperative digital anteroposterior imaging using a standardized technique. To produce the deep pelvic radiograph, a positioning splint with 20° internal rotation of hip joints was used to achieve a standardized and reproducible image during follow-up. X-ray tube was positioned in 1-m distance to the table in perpendicular position. Magnification error was addressed using a ball with known diameter as scaling factor or the known diameter of prosthetic femoral head as an internal reference.

To determine stem tilt and axial stem subsidence, EBRA-FCA (Einzel-Bild-Roentgen-Analyse; University Innsbruck, Austria) was used [21]. A total of 19 reference points were defined on the femoral head (6), the stem (3), the femoral cortex (8), and one at the greater and lesser trochanter, respectively. These reference points define predetermined distances, which are compared by the EBRA-FCA software to calculate implant migration. Radiographs with significant positioning artefacts were excluded by the EBRA-FCA software.

For the EBRA-FCA measurements, a series of at least three radiographs was needed. Thus, radiographs during hospital stay as well as after 6, 12, and 24 months were analyzed. For radiological follow-up, including bone resorption and cortical alterations, only radiographs after surgery and after 24 months were considered.

**Fig. 2:**
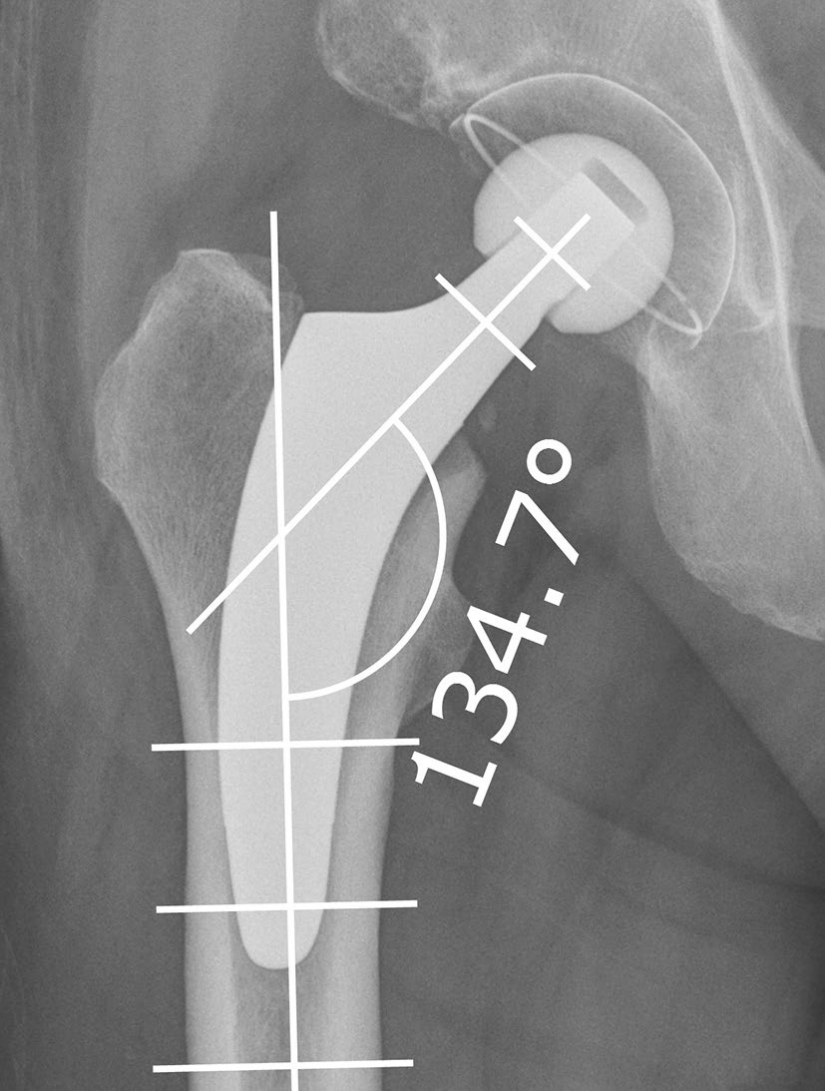
Measurement of CCD angle (between femoral axis and mid-neck-axis of the stem) on postoperative radiographs using the digital templating software MediCAD (Hectec, Landshut, Germany)

In the postoperative radiograph, CCD angles (between femoral axis and mid-neck axis of the stem) were measured retrospectively, using the digital templating software MediCAD (Hectec, Landshut, Germany; Version 3.5) (Fig. 2). Patients were divided into groups A–E regarding different CCD angles [<124.9° (A); 125°–129.9° (B); 130°–134.9° (C); 135°–139.9° (D); >140° (E)]. The rate of stem tilt and axial subsidence, the occurrence of stress-shielding, and cortical hypertrophy, as well as clinical results were analyzed for each group 2 years postoperatively.

**Fig. 3:**
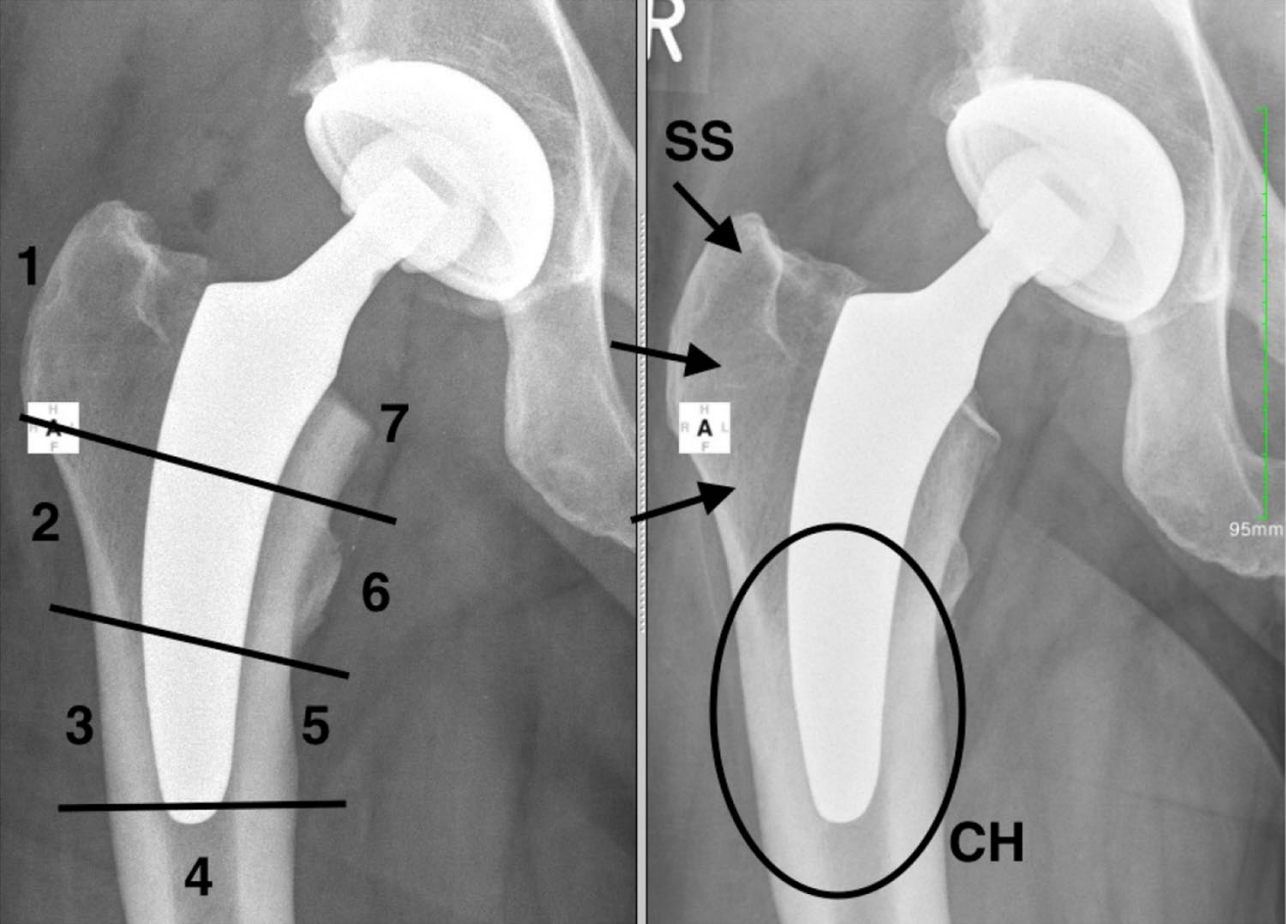
Analysis of stress-shielding and cortical hypertrophy. *Left* modification of the Gruen-zones; *right* 2-year follow-up. Stress-shielding (SS) in Gruen-zone 1 and cortical hypertrophy (CH) in Gruen-zone 3 and 5

Using a modification of the zones described by Gruen [22], bone resorption (stress-shielding) and cortical hypertrophy were analyzed in the standardized radiograph after 2 years (Fig. 3). To detect bone resorption, proximal femoral bone was scanned to find areas with enhanced bone-transparency and thinned or resorbed trabeculae according to the Singh-Index [23]. Grade 1–3 were considered to be stress-shielding. Alterations in periprosthetic cortical bone were analyzed in both radiographs. The increase of cortical width was considered cortical hypertrophy.

In addition, HHS was assessed in all patients after 2 years as well as the rate of periprosthetic fractures.

Mean follow-up time was 2.2 years (range 2.0–3.0 years). Ten hips were either lost to follow up or the EBRA-FCA software failed to accept the radiograph and three patients (4 hips) had deceased unrelated to the operation with prosthesis in situ. In one patient, only a clinical follow-up could be performed. In total, 201 hips in 66 female and 84 male patients fulfilled the inclusion criteria (Table 1).

**Table 1:** Patient’s demographics

CCD category	Demographics				
	Age	Weight	BMI	Gender (w/m)	Unilateral/bilateral
A					
*n*	14			5/9	10/4
Mean (SD)	65.2 (12.5)	80.7 (16.7)	27.6 (5.9)		
95% CI	58.0, 72.4	71.1, 90.4	24.1, 31.0		
Median	69.6	82	27		
Range	33–80	53–111	19–40		
B					
*n*	45			21/24	29/16
Mean (SD)	62.4 (9.9)	80.7 (12.4)	27.1 (4.4)		
95% CI	59.5, 65.4	77.0, 84.4	25.8, 28.4		
Median	62.9	81	27		
Range	40–81	57–115	21–40		
C					
*n*	87			45/42	43/44
Mean (SD)	64.0 (8.9)	82.1 (16.1)	27.6 (4.4)		
95% CI	62.1, 65.9	78.7, 85.6	26.6, 28.5		
Median	63.4	80	27		
Range	36–87	55–153	21–42		
D					
*n*	42			14/28	15/27
Mean (SD)	61.0 (9.1)	89.5 (20.3)	29.5 (6.4)		
95% CI	58.2, 63.9	83.1, 95.8	27.5, 31.5		
Median	60.2	85.5	27		
Range	36–77	50–140	20–45		
E					
*n*	13			2/11	2/11
Mean (SD)	62.7 (8.0)	87.8 (17.8)	27.2 (4.6)		
95% CI	57.9, 67.6	77.0, 98.5	24.4, 29.9		
Median	61	83	27		
Range	54–77	60–125	21–37		
Total					
*n*	201			87/114	51/99
Mean (SD)	63.0 (9.4)	83.6 (16.7)	27.8 (5.02)		
95% CI	61.7, 64.3	81.3, 85.9	21.7, 28.5		
Median	63.0	82.0	27.0		
Range	33–87	50–153	19–45		

*n* number of cases, *SD* standard deviation, *95% CI* 95% confidence interval

For statistical evaluation of stem tilt and subsidence, the last follow-up record was used. Differences between groups were examined non-parametrically using Wilcoxon-two-sample-tests and Kruskal–Wallis test, respectively (i.e., in case of more than two groups). Correlations between subsidence and CCD postoperative were evaluated using Pearson correlations supplemented by Spearman rank correlations, the latter to evaluate the effect of extreme observations. In addition, regression analyses were carried out relating subsidence to CCD postop together with age and weight as potential covariates. To this end, robust regression was applied to account for extreme observations. Associations between CCD angle categorizations and discrete variables (such as stress-shielding occurrence) were examined by Chi-square tests.

For statistical significance, a *p* value of less than 0.05 was considered. The SAS software 9.4 was used for all analyses (SAS Institute, Cary, NC, USA).

## Results

Postoperative range of CCD angles was 117.9°–145.6° with a mean of 132.5° (SD 4.9). Patients were divided into five groups (A–E) regarding postoperative stem alignment measured by CCD angles. Mean postoperative CCD angles for each group were (A) 123.3° (SD 1.8); (B) 128.0° (SD 1.3); (C) 132.4° (SD 1.4); (D) 137.5° (SD 1.4); and (E) 142.5° (SD 2.1), respectively (Table 2).

**Table 2:** Rate of axial subsidence (mm) and varus-/valgus tilt (°) measured in different CCD categories (A–E)

CCD category	*n*	Axial subsidence					Varus/valgus tilt				
		Mean	SD	Median	95% CI		Mean	SD	Median	95% CI	
A (7.0%)	14	−1.20	1.81	−0.65	−2.25	−0.15	−0.16	3.26	−0.15	−2.05	1.72
B (22.4%)	45	−1.02	1.21	−0.70	−1.38	−0.65	0.37	2.72	−0.20	−0.44	1.19
C (43.3%)	87	−1.44	1.34	−1.20	−1.72	−1.15	0.48	2.28	0.50	−0.01	0.96
D (20.9%)	42	−1.50	1.30	−1.35	−1.91	−1.10	0.01	2.94	−0.20	−0.90	0.93
E (6.5%)	13	−2.62	2.17	−2.20	−3.93	−1.30	0.86	2.70	0.80	−0.77	2.49
Total (100.0%)	201	−1.42	144	−1.20	−1.62	−1.22	0.34	2.61	0.20	−0.03	0.70
		Kruskal–Wallis test (*df* = 4): *p* = 0.043					Kruskal–Wallis test (*df* = 4): *p* = 0.502				

Mean varus/valgus stem tilt after 2 years was (A) −0.16° (SD 3.26); (B) 0.37° (SD 2.72); (C) 0.48° (SD 2.28); (D) 0.01° (SD 2.94); and (E) 0.86° (SD 2.70), respectively (Table 2). There was no evidence for significant differences between the groups (*p* = 0.502). Mean axial subsidence in the investigated groups after 2 years summed up to (A) 1.20 mm (SD 1.81); (B) 1.02 mm (SD 1.21); (C) 1.44 mm (SD 1.34); (D) 1.50 mm (SD 1.30); and (E) 2.62 mm (SD 2.17), respectively. Pearson as well as Spearman Correlation showed that the higher the CCD angles are the more pronounced the subsidence was. Regression analysis relating axial subsidence to CCD category together with age and weight as potential covariates accounted for 6.4% of the variation. Age and weight as covariates turned out to be significant. Axial subsidence increased by about 0.26 mm for a 10 year age increase and by about 0.13 mm for a 10 kg weight increase.

The rate of radiological alterations, such as bone resorption and cortical hypertrophy, is summarized in Table 3. Rates in total are low and there was no statistically significant difference found in any of the categories (*p* = 0.220 and *p* = 0.757, respectively).

**Table 3:** Stress-shielding, cortical hypertrophy, and Harris Hip Score analyzed in different CCD categories (A–E)

CCD category	*n*	Stress-shielding	Cortical hypertrophy	Harris Hip Score				
				Mean	Stdev	Median	95% CI	
A (7.0%)	14	0 (0.0%)	0 (0.0%)	96.79	9.23	100	91.45	102.10
B (22.4%)	45	1 (2.2%)	2 (4.4%)	98.09	4.57	100	96.72	99.46
C (43.3%)	87	4 (4.6%)	3 (3.4%)	98.60	3.48	100	97.86	99.34
D (20.9%)	42	1 (2.4%)	3 (7.1%)	97.83	4.81	100	96.33	99.33
E (6.5%)	13	2 (15.4%)	1 (7.7%)	97.92	2.53	99	96.39	99.45
Total (100.0%)	201	8 (4.0%)	9 (4.5%)	98.15	4.55	100	97.52	98.79
		Chi-square-statistics (*df* = 4) *p* = 0.220	Chi-square-statistics (*df* = 4) *p* = 0.757	Kruskal–Wallis test (*df* = 4): *p* = 0.458				

Clinical results, assessed using the HHS, showed no evidence of a difference between different groups after 2 years. Mean HHS in the investigated groups were (A) 96.8 (SD 9.2); (B) 98.1 (SD 4.6); (C) 98.6 (SD 3.5); (D) 97.8 (SD 4.8); and (E) 97.9 (SD 2.5), respectively (*p* = 0.458) (Table 3).

No periprosthetic fractures occurred and none of the stems had to be revised in the observation period.

## Discussion

This study analyzed the outcome of extensive varus- and valgus stem alignment of a calcar-guided short stem regarding implant stability and radiological alterations in a 2-year follow-up. While pronounced femoral varus alignment does not cause increased instability in terms of varus/valgus tilt and axial subsidence, extensive valgus positioning is followed by significantly increased initial subsidence.

**Fig. 4:**
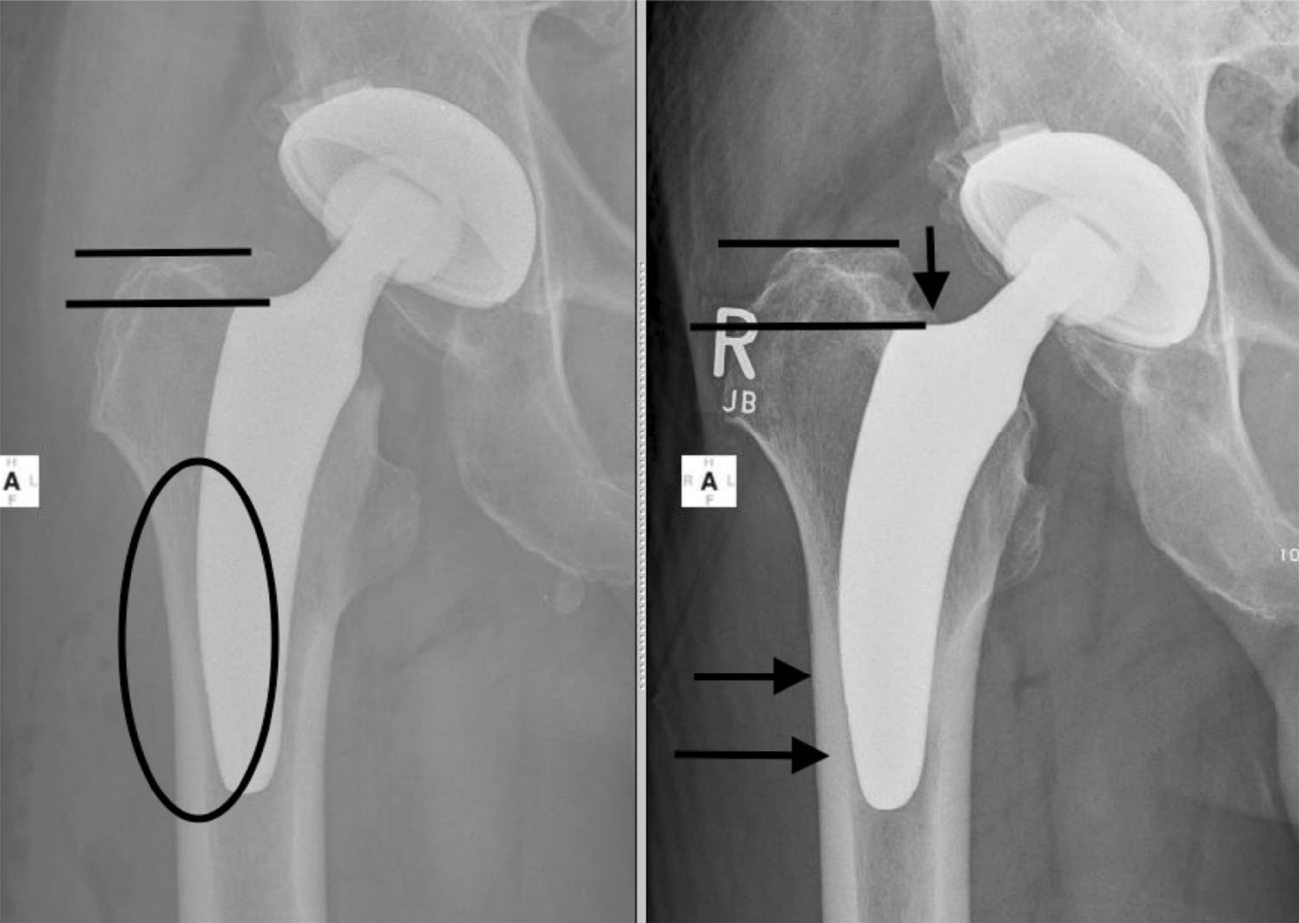
“Undersizing” accompanied with a lack of contact to the lateral cortex, especially in valgus hips, might support initial instability with subsequent implant micromovement

The philosophy of calcar-guided short stems emphasizes on the alignment of the stem alongside the medial calcar in different varus- and valgus position, according to the patient’s anatomy, using individual levels of neck resection [14, 15]. In a comparison of a modular short stem and a conventional straight stem, Schmidutz et al. found a significantly wider range of stem alignment for the short stem (6.2° varus to 8.8° valgus) than for the conventional stem (2.6° varus to 3.3° valgus) [17]. In 24%, stems were implanted in pronounced varus position; in 18%, they were placed in pronounced valgus position [17]. The present collective confirms a broad range of postoperative CCD angles using the investigated implant (Table 1). To position the stem individually, the level of neck resection has to be adapted accordingly. A high resection leads the stem alongside the calcar in a varus position; a valgus position is achieved by deep resection [14, 15]. However, the effect of extensive varus- and valgus alignment on implant migration following short calcar-guided stems has not been investigated. Aseptic loosening is considered to be the most common reason for implant revision [24]. In this context, primary stability, as well as the design specific potential to maintain proximal femoral bone stock is of great importance to predict implant failure [6]. For fixation of metaphyseal anchoring short stems, a sufficient cortical ring is recommended [5]. In varus anatomy, given a high neck resection, ring-fixation is accentuated. However, in valgus anatomy, given the need of a low resection level to place the stem in the intended valgus position, the ring-fixation might be compromised [5] with subsequent varus- or valgus tilt and axial subsidence after full weight bearing. Therefore, in addition, sufficient contact of the tip of the stem to the lateral cortex in regard to achieving a classical three-point anchoring is of great importance (Fig. 4). Again, in pronounced varus stem alignment, contact of the tip of the stem to the lateral cortex is commonly achieved. In valgus position, however, a missing cortical contact of the tip can be frequently observed, particularly in cases of “undersizing” (Fig. 4). Hence, the operating surgeon should be aware, that “undersizing” accompanied with a lack of contact to the lateral cortex, especially in valgus hips, might support initial instability with subsequent implant micromovement. Due to the different implant design of the investigated stem, in contrast to the Metha stem, in those cases, a resection to the level of the fossa piriformis should be pursued, to implant the adequate size of the stem and avoid “undersizing”. Earlier published results on the migration pattern of the investigated short stem showed a significant influence of body weight on the rate of early axial subsidence [19]. The regression analysis relating axial subsidence to CCD categories confirms the impact of heavy weight accordingly in the present investigation. However, the model accounted for only 6.4% of the variation.

Furthermore, it remains unclear how different resection heights and stem alignments affect load transfer and stress-shielding patterns, which are, besides micromovement of the implant, also generally thought to have a considerable effect on aseptic loosening [5]. Lerch et al. found no correlation between stem alignment, stem size, or resection level with changes in bone mineral density (BMD) in a recent investigation of the Metha short stem (Aesculap, Tuttlingen, Germany) using dual-energy X-ray absorptiometry (DEXA) [25]. However, the small size of the study population might not allow valid conclusions. Floerkemeier et al., in a biomechanical study using strain gauges on synthetic composite femora, measuring strain patterns of three different resection levels after implanting also the Metha short stem (Aesculap, Tuttlingen, Germany), found a decrease in strain in the region of the greater trochanter by 30, 32, and 52% for the high-, medium-, and low-neck resection level, respectively [5]. This corresponds to the results of this study, with stems aligned in extensive valgus position showing slightly enhanced stress-shielding in Gruen-zone 1, compared to implants in varus position, although not being statistically significant. In Gruen-zone 7, the medial calcar cortex, the biomechanical measurements resulted in slightly reduced strains for the low- and medium-resection level, while there was a strain increase of 43% for the high-resection level [5]. Again, this study confirms these findings, showing a slightly pronounced bone resorption on the medial part of the neck in valgus hips with low resection level, compared to varus hips with high-resection level, however, without any statistical significance.

Aligning the stem in an extensive varus position results in an increase in offset and subsequent enhanced lever-arm compared to the extensive valgus alignment, in turn, leading to an increased strain in the medial part of the proximal femur and the region around the distal tip of the stem [5]. Given increased forces, especially on the medial cortical bone, with some stem designs, a higher risk of intraoperative and postoperative periprosthetic fractures has been reported [9]. However, after 2 years in the present investigation, no periprosthetic fractures were observed. Theoretically, abnormal load distribution might lead to cortical hypertrophy in Gruen-zones 3 and 5 with possible subsequent thigh pain [10, 26]. In a recent study, analyzing the Fitmore stem (Zimmer, Warsaw, IN, USA), cortical hypertrophy was observed in 63% of hips accompanied with a low rate of thigh pain, implying distal loading and proximal stress bypass [27]. In contrast, the optimys stem is designed with a polished tip to retain strain peaks in the distal part and, therefore, avoid cortical reactions. The overall incidence after a follow-up of 2 years has been shown to be below 5% without any appearance of thigh pain or other clinical consequences [28]. A slight emphasis regarding cortical hypertrophy could be found for valgus hips, however, not being statistically significant. The small number of cortical hypertrophy in the present study does not allow valid conclusions regarding the impact of different extensive stem alignment.

This study has several limitations. First to be mentioned is the short follow-up of 2 years. Although only long-term results should be considered valid, the initial evaluation of bony alterations is necessary to identify undesirable results [29]. Early migration analysis may allow a prediction of implant survival [7] and the reaction of environmental bone in the early stage may help to predict the long-term outcome. Second, RSA provides higher accuracy in comparison to the EBRA-FCA method used in this study. The computer-assisted EBRA-FCA system was evaluated to be able to detect stem subsidence of ±1 mm and varus/valgus tilting of ±0.4° given a specificity of 100% and sensitivity of 78% [21]. However, the migration pattern after 2 years has been established in several studies using EBRA-FCA providing a reference to long-term survival [7, 30]. Furthermore, measuring the CCD angles in 2D radiographs lacks some accuracy compared to 3D imaging. However, radiographs were done using a standardized protocol to reduce a possible bias.

## Conclusions

The results confirm a wide range of CCD angles to be adequately reconstructed using the investigated calcar-guided short stem. After 2 years, extensive varus positioning does not cause increased instability in terms of varus/valgus tilt and axial subsidence suggesting no restrictions regarding indications. Extensive valgus positioning is followed by significantly increased subsidence without any clinical correlation. Undersizing the stem providing insufficient cortical contact could be identified as the main cause leading to axial subsidence, especially in extensive valgus alignments. The overall rate of stress-shielding and cortical hypertrophy is low without any clinical consequences. Short-term clinical results in all groups are encouraging. Further monitoring, especially of the valgus hips, is mandatory.
